# Supplementary material for: Aconitate Decarboxylase 1 Deficiency Exacerbates Mouse Colitis Induced by Dextran Sodium Sulfate
Source: Int J Mol Sci. 2022 Apr 15;23(8):4392. doi: 10.3390/ijms23084392 (PMC9025264; doi:10.3390/ijms23084392)
Supplement: Supplementary file 1 [file ijms-23-04392-s001.zip › ijms-1626097-supplementary.pdf]

Supplemental Figure Legends

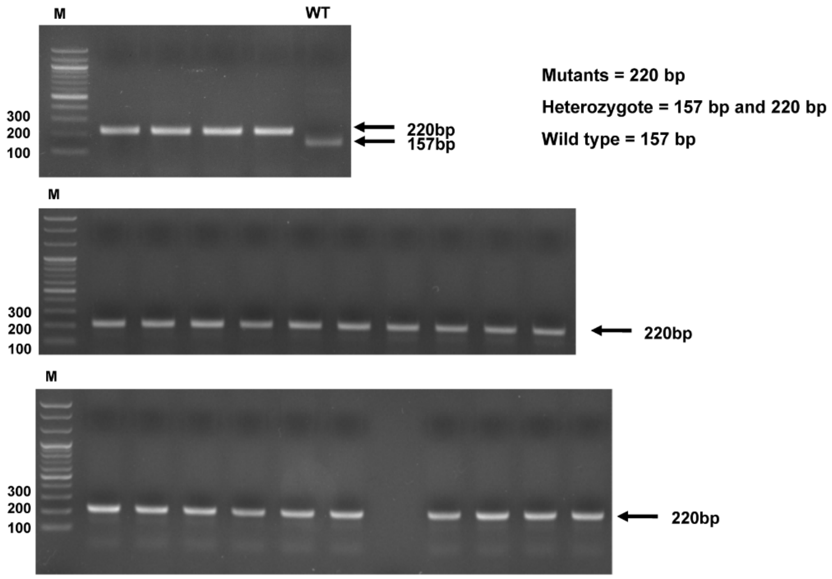

Supplemental Figure S1. Genotyping of C57BL/6NJ-*Acod1*$^{em1(IMPC)J}$/J. 220 bp band means mutant, and 157 bp band means wild-type. Both bands indicate heterozygote upon detection.

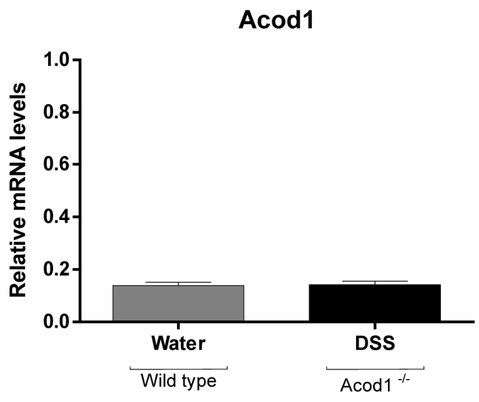

Supplemental Figure S2. The colonic mRNA levels of Acod1 in *acod1*$^{-/-}$ mice treated with DSS. The mRNA levels of Acod1 in the colonic tissues were determined by quantitative real time-PCR.
